# Association between maternal overprotection and premenstrual disorder: a machine learning based exploratory study

**DOI:** 10.1186/s13030-025-00326-y

**Published:** 2025-02-24

**Authors:** Kaori Tsuyuki, Miho Egawa, Takuma Ohsuga, Akihiko Ueda, Kazuki Shimada, Tsukasa Ueno, Kazuko Hiyoshi, Keita Ueda, Masaki Mandai

**Affiliations:** 1 Department of Gynecology and Obstetrics, Kyoto University Graduate School of Medicine, Kyoto, Japan; 2 Integrated Clinical Education Center, Kyoto University Hospital, Kyoto, Japan; 3 Department of Psychiatry, Kyoto University Graduate School of Medicine, Kyoto, Japan; 4 Department of Nursing, Taisei Gakuin University, Osaka, Japan; 5 Department of Medical and Social, Faculty of Health Science, Kyoto Koka Women’s University, Kyoto, Japan

**Keywords:** Premenstrual syndrome, Premenstrual dysphoric disorder, Premenstrual disorder, Machine learning analysis, Parental bonding, Overprotection, Affective vulnerability

## Abstract

**Background:**

Premenstrual disorder (PMD), which includes premenstrual syndrome and premenstrual dysphoric disorder, has a complex pathogenesis and may be closely related to emotional cognition and memory. However, the mechanisms underlying these associations remain unclear. Therefore, this study used machine learning to explore the roles of various factors that are not typically considered risk-factors for PMD.

**Methods:**

A predictive model for PMD was constructed using a dataset of questionnaire responses and heartrate variability data collected from 60 participants during their follicular and luteal phases. Based on the Japanese version of the Premenstrual Symptom Screening Tool, the binary objective variable (PMD status) was defined as “PMD” for moderate-to-severe premenstrual syndrome and premenstrual dysphoric disorder and other conditions as “non-PMD.” The contribution of each feature to the predictive model was assessed using the Shapley Additive exPlanations (SHAP) model-interpretation framework.

**Results:**

Of the 58 participants (providing 117 data points), 17 (34 data points) were in the PMD group and 41 (83 data points) were in the non-PMD group. The area under the receiver operating characteristic curve was 0.90 (95% confidence interval: 0.82–0.98). Among the top 20 features with the highest SHAP values, six were associated with maternal bonding. Four of the six mother-related characteristics were associated with overprotection.

**Conclusions:**

Based on these findings, parental bonding experiences, including maternal overprotection, may be associated with the presence of PMD.

**Supplementary Information:**

The online version contains supplementary material available at 10.1186/s13030-025-00326-y.

## Background

Premenstrual syndrome (PMS), which occurs three to ten days before every menstruation [1], is experienced by approximately half of women of reproductive age [2]. PMS exhibits a wide range of symptoms, the most common being breast tenderness, bloating, headaches, mood swings, depression, anxiety, anger, and irritability. Premenstrual dysphoric disorder (PMDD) is a severe form of PMS with predominantly psychiatric symptoms. PMDD was first mentioned as a research criterion in the 1994 Diagnostic and Statistical Manual of Mental Disorders (DSM)-IV of the American Psychiatric Association [3]. In DSM-5, the disorder is presented as a diagnostic criterion and is listed as a depressive disorder [4]. PMS and PMDD are now referred to as premenstrual disorder (PMD) [5, 6]. Women suffering from PMD experience substantial difficulties related to social activities and interactions.

PMD has various treatment options, including pharmacological therapies such as selective serotonin reuptake inhibitors and non-pharmacological approaches like cognitive behavioral therapy and regular exercise [7–14]. However, these treatments are not universally effective, necessitating a personalized approach based on individual causes and symptom profiles.

PMD exhibits a complex pathophysiology involving central neurotransmitters, ovarian hormones, and neurosteroids [15]. Smoking, alcohol use, obesity, and nutrient deficiencies are known risk-factors for PMD [16, 17], and psychosocial factors, such as childhood trauma and sexual abuse, have been reported to be associated with PMD [18–20]. Although various risk factors for PMD are well established, other factors that have not traditionally been recognized as risk factors, including subtle aspects of emotional regulation, may also contribute to its development. Fluctuations in emotional cognition and memory are associated with the menstrual cycle [21–23] and may be associated with the expression of PMD. Nonetheless, the mechanisms underlying these interactions remain unclear.

Machine learning, a subfield of artificial intelligence, has emerged as a promising tool for addressing various challenges in clinical medicine [24]. In particular, the development of interpretable machine learning techniques, such as Shapley Additive Explanations (SHAP), has advanced the application of machine learning in healthcare, overcoming previous limitations related to explainability [25]. Given that PMD is a highly multifactorial condition, the complexity of interactions among its contributing factors suggests that machine learning approaches provide a suitable methodology for elucidating these intricate relationships. Utilizing machine learning, this study aimed to examine the effects of these less-studied variables. For this purpose, this study utilized data obtained in prior studies of brain function, the autonomic nervous system, and the menstrual cycle (under analysis). Investigating these factors could reveal unexplored pathways associated with PMD, potentially leading to innovative interventions that improve quality of life by promoting more comprehensive management strategies.

## Methods

### Study design and setting

This cross-sectional study recruited 60 women at Kyoto University (Kyoto, Japan) between December 2018 and April 2021. Women of at least 18 years of age and younger than 45, with regular menstrual cycles, were included. The participants were female university and graduate students as well as female workers. Recruitment was conducted via posters and flyers displayed and distributed at the university. The exclusion criteria included: (a) Women with secondary amenorrhea, in which menstruation has stopped for more than three months, including physiological amenorrhea during pregnancy or lactation and medical menopause due to gynecological surgery; (b) women with oligomenorrhea, in which the menstrual cycle exceeds 39 days; and (c) women taking hormonal drugs such as low-dose estrogen/progestin oral contraceptives. As the study involved magnetic resonance imaging, women who experienced claustrophobia, those with tattoos, and those with metal objects (e.g., pacemakers) in the body were also excluded.

### Data set

**Fig. 1:**
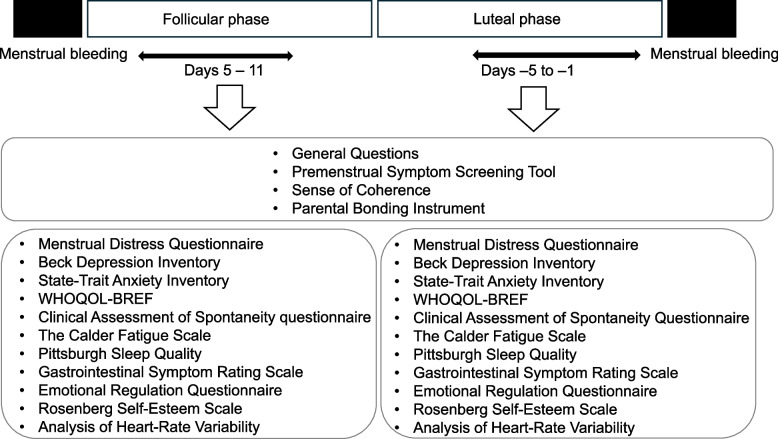
Study overview. The participants were examined twice, once during the follicular phase (within five to ten days after the onset of menstrual bleeding) and once during the late-luteal phase (within five days before the next menstruation). For each participant, the data from each phase were counted as one data point. In total, 121 data points were obtained from 60 participants. General questions, the Premenstrual Symptom Screening Tool, Sense of Coherence (SOC), and the Parental Bonding Instrument (PBI) were completed once; the other parts of the questionnaires were completed at both the follicular and late-luteal phases. All responses were converted to numerical or categorical variables, and free descriptions were removed. Spectral analysis of heart-rate variability (HRV) was performed for the follicular and late-luteal phases. To obtain the binary objective variable, the Japanese version of the Premenstrual Symptom Screening Tool (PSST) was applied, placing participants with moderate-to-severe premenstrual syndrome (PMS) and premenstrual dysphoric disorder (PMDD) in the “PMD” group and those with other conditions in the “non-PMD” group. The explanatory variables comprised 443 features. PMD, Premenstrual disorder

The participants were examined on two separate occasions: once during the follicular phase (within five to ten days after the onset of menstrual bleeding) and once during the late-luteal phase (within five days before the next menstruation). The timing of the late-luteal phase was determined by predicting and measuring the next menstrual period and then obtaining the date of the next menstrual onset, thereby confirming that it was within this timeframe. The study flowchart is presented in Fig. 1. For each participant, the data from each phase were counted as one data point. An objective variable and explanatory variables were derived using this dataset, and missing values were imputed using the means for all participants.

### Objective variable

PMD status, the binary objective variable, was assessed using the Japanese version of the Premenstrual Symptom Screening Tool (PSST), a screening tool for moderate-to-severe PMS/PMDD [26] based on the DSM-IV [3]. Using this approach, participants with moderate-to-severe PMS and PMDD were assigned to the “PMD” group and those with other conditions to the “non-PMD” group.

### Explanatory variables

The questionnaire comprised thirteen parts: General questions (regarding age, marital status, education, menstrual history, pregnancy history, and sleep duration); Sense of Coherence (SOC) [27]; Parker’s Parental Bonding Instrument (PBI) [28]; the Menstrual Distress Questionnaire [29]; the Beck Depression Inventory [30]; the State-Trait Anxiety Inventory [31]; the Japanese version of the World Health Organization Quality-Of-Life Scale [32]; Clinical Assessment of Spontaneity questionnaire [33]; the Chalder Fatigue Scale [34]; Pittsburgh Sleep Quality Index [35]; Gastrointestinal Symptom Rating Scale [36]; Emotional Regulation Questionnaire [37]; and the Rosenberg Self-Esteem Scale [38]. Answers to the general questions, SOC, and PBI were collected once, whereas answers to other questions were collected at both the follicular and late-luteal phases. All responses were converted to numerical or categorical values and free descriptions were removed. To assess autonomic nervous activity, spectral analysis was performed on heartrate variability (HRV) in the follicular and late-luteal phases. This was done by measuring the R–R interval during orthostatic loading (from sitting to standing to sitting), with electrocardiograms obtained via electrodes on both wrists. HRV was analyzed using the time-domain analysis method, which utilizes the coefficient of variation of the R–R interval. Variation in R–R intervals was separated by frequency, and the categories ‘Low Frequency’ and ‘High Frequency’ were used to simultaneously quantify sympathetic and parasympathetic nervous system activities. A Kiritsu Meijin autonomic reflex orthostatic tolerance recorder (Crosswell Inc., Yokohama, Japan) was utilized. Together, the questionnaires and HRV analyses generated 443 explanatory variable features (Additional File 1).

### Construction of the PMD-prediction model

Using this dataset, a PMD-prediction model was constructed. DataRobot (SaaS, DataRobot, Tokyo, Japan) was used to search for appropriate machine learning algorithms and a Random Forest Classifier was selected to build the predictive model. Overall model performance was evaluated based on the area under the receiver operating characteristic curve (AUC). The model was then reconstructed using Recursive Feature Elimination to maximize AUC by searching for the optimal number of features among the 443 explanatory variables. Stratified five-fold cross validation was used to validate the model. The contribution of each feature to the model was assessed using Shapley Additive exPlanations (SHAP), a game-theory-based model-interpretation framework that quantitatively evaluates the contribution of each input feature as a SHAP value [25]. The SHAP method was implemented using the Python SHAP package. The analyses were performed in Python 3.8.2 using scikit-learn 1.1.1.

### Statistical analysis

Statistical analyses were conducted using Python 3.8.2, with scipy.stat. Background characteristics are presented as proportions (%) or means (standard deviation). Statistical tests were conducted on the features with high SHAP values based on the machine-learning analysis. The normality of the variables was examined using the Shapiro–Wilk test. For two-group comparisons, the Mann–Whitney *U*-test was used for non-normally distributed variables and Welch’s *t*-test for normally distributed variables.

## Results

### Participant characteristics

All of the 60 participants assessed were eligible for inclusion in the study and provided informed consent. None of the participants chose to withdraw from the study. In total, 121 luteal or follicular phases were observed (twice in the luteal phase for one person). Two participants, with missing PSST responses, were excluded during data analysis. The remaining 58 participants provided 117 data points, and 17 (34 data points) were assigned to the PMD group and 41 (83 data points) to the non-PMD group.

Among the 58 participants, the General Questions, PSST, SOC, and PBI were administered to 32 participants during the late-luteal phase and to 24 during the follicular phase. One participant completed the questionnaires at a time that did not correspond to either the late-luteal or follicular phase, and the timing of administration for another participant was missing. The characteristics of the participants in each group are presented in Table 1.

**Table 1:** Background characteristics of the participants in the premenstrual disorder (PMD) and non-PMD groups

**Characteristic**	**PMD group (*n* = 34)**		**non-PMD group (*n* = 83)**	
	*n* or Mean	% or SD	n or Mean	% or SD
Age (years)	28,8	8,3	29,1	8
Age at first menstruation (years)	12,3	1,2	12,5	1,3
*Marital status*				
Never married	24	70	69	83,1
Married	10	30	14	16,8
Divorced, widowed, other	0	0	0	0

*SD* Standard Deviation

### PMD-prediction model

**Fig. 2:**
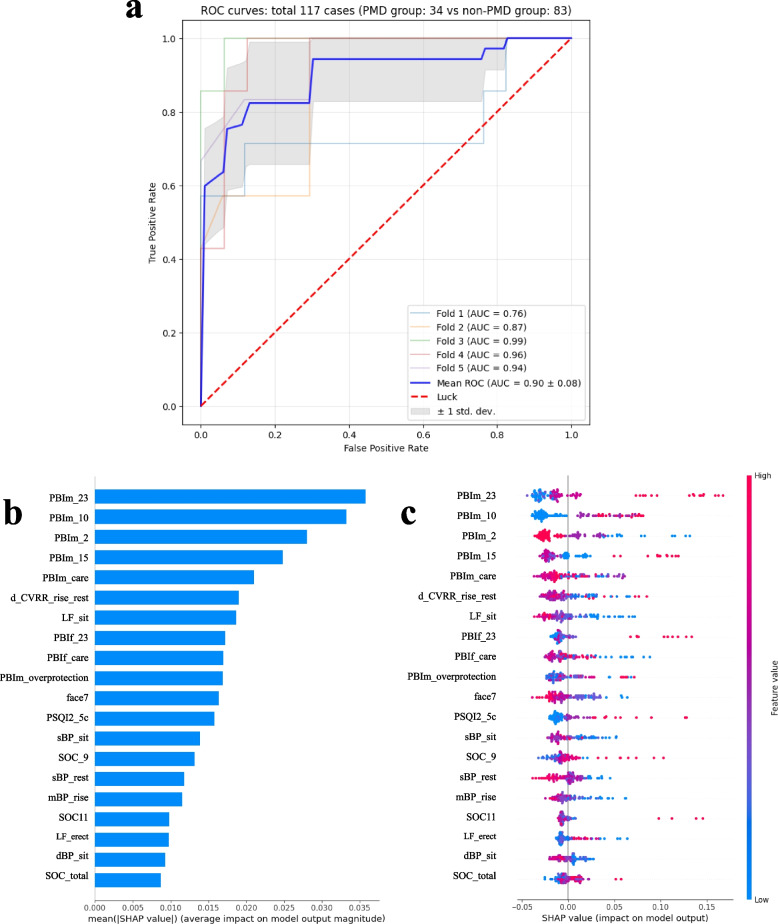
Visualization of model performance and contributing features. **a** Model performance, based on the area under the receiver operating characteristic (ROC) curve. **b** Features with higher overall Shapley Additive exPlanations (SHAP) scores, indicating higher average contributions to premenstrual disorder (PMD) prediction. **c** Positive and negative contributions to PMD, characterized by positive and negative SHAP values, respectively. Red and blue represent the magnitude of the values. AUC, area under the receiver operating characteristic curve; PBIm/f, Parental Bonding Instrument (mother/father); MDQ, Menstrual Distress Questionnaire; PSQI, Pittsburgh Sleep Quality Index; BP, blood pressure (s, systolic; d, diastolic; m, mean); SOC, Sense of Coherence; STAI, State–Trait Anxiety Inventory. Items PBIm_23, PBIm_10, and PBIf_23 are scored as follows: very like, 3; moderately like, 2; moderately unlike, 1; and very unlike, 0. Items PBIm_2 and PBIm_15 were reverse scored. The PBIm_care and PBIf_care scores range from to 0–36. The PBIm_overprotection scores range from to 0–39. For information on the other measures, see Additional file 1

Among the 443 features, Recursive Feature Elimination identified 31 that are potentially associated with PMD. The relation between the number of features and the AUC score is shown in the Figure in Additional File 2 and the selected features are listed in Additional File 3. The generalization performance of the model estimated based on the test data had an AUC of 0.90 (95% confidence interval, 0.82–0.98) (Fig. 2a). The top 20 features with the highest SHAP values are shown in Fig. 2b and c: six are associated with PBI items related to mothers (PBIm) and two with PBI items related to fathers (PBIf). Four of the six mother-related characteristics are associated with overprotection.

### PBI factors contributing to PMD

**Fig. 3:**
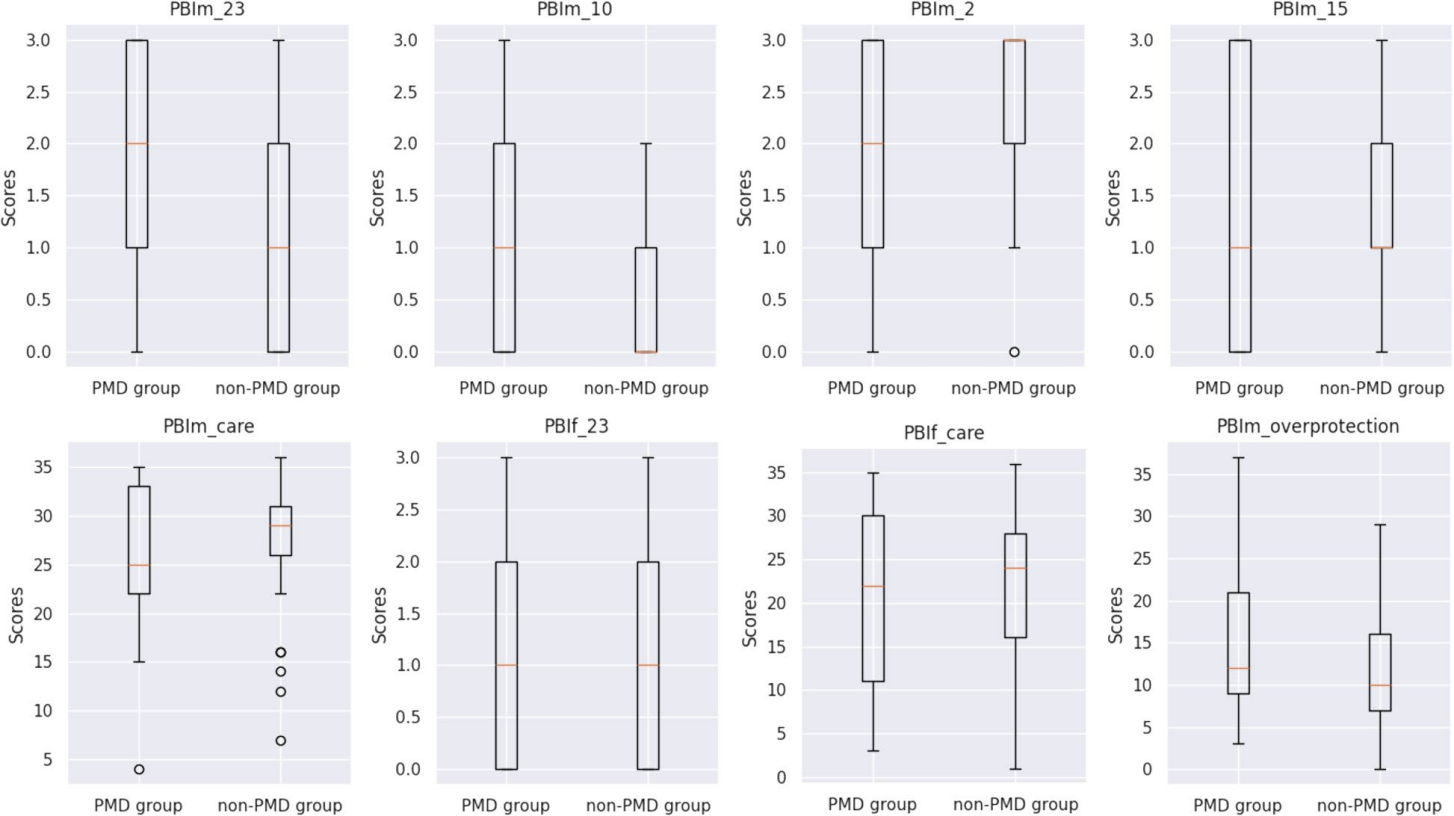
Parental Bonding Instrument (PBI) questionnaire factors contributing to premenstrual disorder (PMD). The Mann–Whitney *U*-test was used to analyze PBIm_23, PBIm_10, PBIm_2, PBIm_15, PBIm_care, PBIf_23, and PBIm_overprotection; Welch’s *t*-test was used to analyze PBIf_care. PBIm/f, PBI items related to mother/father

Through analysis of the complete PSST data of 58 participants with complete PSST data,; 29% (*n* = 17) were assigned to the PMD group and 71% (*n* = 41) to the non-PMD group. Among the top 20 features with the highest SHAP values, eight (related to items in the PBI questionnaire) were compared based on PMD status (Fig. 3, Table 2). The scores for specific items varied notably between the PMD and non-PMD groups. For instance, for the item “Was overprotective of me” (PBIm23), the median (IQR) scores for the PMD and non-PMD groups were 2 (1–3) and 1 (0–2), respectively (*p* = 0.008); similarly, for “Invaded my privacy” (PBIm10), these scores were 1 (0–2) and 1 (0–1), respectively (*p* = 0.008). In contrast, for “Did not help me as much as I needed” (PBIm2), these scores were 2 (1–3) for PMD and 3 (2–3) for non-PMD (*p* = 0.012). The total PBI scores for parental overprotection and care did not vary notably between the PMD and non-PMD groups (Additional File 4).

**Table 2:** Parental Bonding Instrument (PBI) questionnaire factors contributing to premenstrual disorder (PMD)

**Factors**	**Questions**	**PMD group (*n* = 17)**				**non-PMD group (*n* = 41)**				**Statistics**	
		Median	IQR	Mean	SD	Median	IQR	Mean	SD	*p*-value	95% CI
**PBIm 23**^**a**^	Was overprotective of me	2	1–3	1	1	1	0–2	1,9	1,1	0,008	-
**PBIm 10**^**a**^	Invaded my privacy	1	0–2	0,4	0,7	0	0–1	1	0,9	0,008	-
**PBIm 2**^**a**^	Did not help me as much as I needed	2	1–3	2	0,7	3	2–3	2,6	0,9	0,012	-
**PBIm 15**^**a**^	Let me decide things for myself	1	0–3	1,2	0,8	1	1–2	1,2	1,3	0,87	-
**PBIm care**^**a**^	Items: 1, 2, 4, 5, 6, 11, 12, 14, 16, 17, 18, 24	25	22–33	25,7	8,3	29	26–31	27,8	6,7		-
**PBIf 23**^**a**^	Was overprotective of me	1	0–2	1,3	1,2	1	0–2	0,9	0,9	0,33	-
**PBIf care**^**b**^	Items: 1, 2, 4, 5, 6, 11, 12, 14, 16, 17, 18, 24	22	11–30	20,8	10,9	24	16–28	22,6	8,6	0,74	(− 7.9, 4.4)
**PBIm overprotection**^**a**^	Items: 3, 7, 8, 9, 10, 13, 15, 19, 20, 21, 22, 23, 25	12	9–21	11,6	6,5	10	7–16	15,4	10,3	0,26	-

*IQR* Interquartile Range, *SD* Standard Deviation, *PBIm/f* PBI items related to mother/father

^a^Mann-Whitney *U*-test

^b^Welch’s *t*-test

## Discussion

This machine learning-based PMD-prediction model applies the novel perspective of SHAP values to the exploration of the association between specific parental-bonding experiences, such as maternal overprotection, and the presence of PMD. To the best of our knowledge, this study is the first to report an association between parental-bonding style and PMD. Among the selected characteristics, a number of factors related to the parent–child relationship were identified. This suggests a considerable association between PMD and the family environment, particularly overprotection.

Parker’s PBI, a 25-item self-report questionnaire, measures how an individual remembers their parents during their first 16 years [28]. Examination of the PBI has shed light on its associations with many health outcomes, including mood disorders, inflammatory bowel disease, and chronic pain [39–42]. Notably, these associations exhibit gender-specific nuances, with maternal influences predominantly affecting women and paternal influences affecting men [40]. When focusing on women’s health, it is therefore understandable that maternal influence is likely to be revealed to be more important than paternal influence.

Considering early-life experiences, parental maltreatment is a known risk factor for PMD. The profound impact of early-life trauma, including both physical and psychological abuse, on the manifestation of PMD has been well-documented [18–20]. Moreover, temperament–personality assessments have shown that damage avoidance and low levels of self-orientation are significantly correlated with the prevalence of PMD [19]. However, our current analysis unexpectedly revealed an association between PMD and ‘overprotection,’ a parent–child relationship dynamic that stands in stark contrast to maltreatment. To contextualize these paradoxical findings, we attempt to interpret their implications within the framework of affective vulnerability and hypersensitivity, concepts that provide valuable insights into understanding PMD. Individuals with PMD may exhibit increased sensitivity to the normal fluctuations in estrogen and progesterone levels during the menstrual cycle [43]. Such women may have a latent affective vulnerability as well as a low threshold for perceiving physical symptoms. The hormonal fluctuations associated with the menstrual cycle may exacerbate these characteristics and interfere in daily life, heightening susceptibility to PMD. This vulnerability may arise from interactions between genetic and environmental factors. Women with such characteristics and dispositions are likely to have difficulty in coping flexibly with various events in their lives, both before and after menarche, and it is natural that mothers may tend to be overprotective of such daughters.

The mother of a daughter with PMD is also likely to be affectively vulnerable, with existing studies suggesting that affective vulnerability could be genetically inherited from parents with mood disorders [44]. While there may be a risk of maltreatment from mothers with mental illness, those who tend to be anxious but below the threshold of illness may tend to behave in an overprotective manner. Consequently, it remains difficult to confirm a causal relationship between parenting style and the cause of PMD.

Notably, the PBI questionnaire results did not differ conclusively between the PMD and non-PMD groups based on classical statistical methods. However, some intergroup differences were observed for specific sub-items, indicating the need for further investigation of these aspects. This supports the multifaceted nature of PMD and that comprehensive approaches that consider both biological and psychosocial factors are necessary for its effective management and prevention.

On the whole, further investigations are required to explore the associations between affective vulnerability and PMD, as well as those between perceptions of parental overprotection, affective vulnerability, and related factors, such as comorbidities, neurodevelopment, and nutritional deficiencies.

This study had several limitations. First, the questionnaire did not cover risk factors for PMD. Questions about experiences of abuse or trauma or about socioeconomic status were not included, making it difficult to compare the relative contributions of the known risk factors and the parental-bonding styles examined in this study.

Second, the number of participants was limited, and the sample was not representative of Japanese women. The participants reside in the same region of Japan; hence, it was presumed that they share comparable social status: these factors could influence both their perceptions and the style of parenting that they experienced. Furthermore, the samples were obtained via convenient and nonrandom methods, which probably affects the generalizability of the results. Therefore, further studies are required.

Third, only one objective variable (PMD status) was examined based on the PSST. The reliability and validity of the Japanese version of the PSST used in this study remains to be fully verified. Moreover, the PSST does not extract all types of PMD. However, the PSST is used globally and has gained recognition as a standard measure of PMS/PMDD.

## Conclusions

This study is the first to report an association between parental-bonding style and PMD, based on a wide range of factors. The results of the machine learning-based predictive model suggest an association between specific parental-bonding experiences, such as maternal overprotection, and the occurrence of PMD. Further research is needed to examine the interactions between the psychosocial factors and biological aspects of the menstrual cycle.

## Supplementary Information

Additional file 1. Explanatory Variable Features. This file contains detailed information on the 443 explanatory variables.

Additional file 2. Searching for the optimal number of features. This file contains the figure used to explore the number of features via recursive feature elimination of the 443 features, to obtain the highest the area under the receiver operating characteristic curve (AUROC).

Additional file 3. Selected 31 features. This file contains information describing the 31 explanatory variables selected by searching for the optimal number of features.

Additional file 4. PBI scores of the PMD and non-PMD groups. This file presents the descriptive statistics of the two groups for the PBI and the results of the Mann–Whitney *U*-test and Welch’s *t*-test, used to compare the groups.

## Data Availability

The datasets analyzed in this study are available from the corresponding author on reasonable request.

## Abbreviations

AUC: Area under the receiver operating characteristic curve
DSM: Diagnostic and Statistical Manual of Mental Disorders
HRV: Heartrate variability
PBI: Parental bonding instrument
PMDD: Premenstrual dysphoric disorder
PMD: Premenstrual disorder
PMS: Premenstrual syndrome
PSST: Premenstrual Symptoms Screening Tool
SHAP: Shapley Additive exPlanations
SOC: Sense of coherence

## References

[1] Yonkers KA, O’Brien PM, Eriksson E. Premenstrual syndrome. Lancet. 2008;371(9619):1200–10. 18395582 10.1016/S0140-6736(08)60527-9 PMC3118460

[2] Direkvand-Moghadam A, Sayehmiri K, Delpiseh A, Sattar K. Epidemiology of premenstrual syndrome (PMS)-A systematic review and meta-analysis study. J Clin Diagn Res. 2014;8(2):106–9. 24701496 10.7860/JCDR/2014/8024.4021 PMC3972521

[3] American Psychiatric Association. Diagnostic and statistical manual of mental disorders. 4th ed. Washington, DC: American Psychiatric Association; 1994.

[4] American Psychiatric Association. Diagnostic and statistical manual of mental disorders. 5th ed. Washington, DC: American Psychiatric Association; 2013.

[5] O’Brien PM, Backstrom T, Brown C, Dennerstein L, Endicott J, Epperson CN, et al. Towards a consensus on diagnostic criteria, measurement and trial design of the premenstrual disorders: The ISPMD Montreal consensus. Arch Womens Ment Health. 2011;14(1):13–21. 21225438 10.1007/s00737-010-0201-3 PMC4134928

[6] Ismaili E, Walsh S, O’Brien PMS, Bäckström T, Brown C, Dennerstein L, et al. Fourth consensus of the International Society for Premenstrual Disorders (ISPMD): Auditable standards for diagnosis and management of premenstrual disorder. Arch Womens Ment Health. 2016;19(6):953–8. 27378473 10.1007/s00737-016-0631-7

[7] Tiranini L, Nappi RE. Recent advances in understanding/management of premenstrual dysphoric disorder/premenstrual syndrome. Fac Rev. 2022;11:11. 35574174 10.12703/r/11-11 PMC9066446

[8] Green LJ, O’Brien PM, Panay N, Craig M, Royal College of Obstetricians and Gynaecologists. Management of premenstrual syndrome: green-top guideline No. 48. BJOG. 2017;124(3):e73–105. 10.1111/1471-0528.14260 27900828

[9] Kancheva Landolt N, Ivanov K. Short report: Cognitive behavioral therapy - a primary mode for premenstrual syndrome management: Systematic literature review. Psychol Health Med. 2021;26(10):1282–93. 32845159 10.1080/13548506.2020.1810718

[10] Pearce E, Jolly K, Jones LL, Matthewman G, Zanganeh M, Daley A. Exercise for premenstrual syndrome: a systematic review and meta-analysis of randomised controlled trials. BJGP Open. 2020;4(3):bjgpopen20X101032. 32522750 10.3399/bjgpopen20X101032 PMC7465566

[11] Abdi F, Ozgoli G, Rahnemaie FS. A systematic review of the role of vitamin D and calcium in premenstrual syndrome. Obstet Gynecol Sci. 2019;62(2):73–86. 30918875 10.5468/ogs.2019.62.2.73 PMC6422848

[12] Abdollahi R, Abiri B, Sarbakhsh P, Kashanian M, Vafa M. The effect of vitamin D supplement consumption on premenstrual syndrome in vitamin D-deficient young girls: a randomized, double-blind, placebo-controlled clinical trial. Complement Med Res. 2019;26(5):336–42. 31104056 10.1159/000500016

[13] Heidari H, Amani R, Feizi A, Askari G, Kohan S, Tavasoli P. Vitamin D supplementation for premenstrual syndrome-related inflammation and antioxidant markers in students with vitamin D deficient: a randomized clinical trial. Sci Rep. 2019;9(1):14939. 31624297 10.1038/s41598-019-51498-x PMC6797739

[14] Moslehi M, Arab A, Shadnoush M, Hajianfar H. The association between serum magnesium and premenstrual syndrome: a systematic review and meta-analysis of observational studies. Biol Trace Elem Res. 2019;192(2):145–52. 30880352 10.1007/s12011-019-01672-z

[15] Rapkin AJ, Akopians AL. Pathophysiology of premenstrual syndrome and premenstrual dysphoric disorder. Menopause Int. 2012;18(2):52–9. 22611222 10.1258/mi.2012.012014

[16] Chocano-Bedoya PO, Manson JE, Hankinson SE, Johnson SR, Chasan-Taber L, Ronnenberg AG, et al. Intake of selected minerals and risk of premenstrual syndrome. Am J Epidemiol. 2013;177(10):1118–27. 23444100 10.1093/aje/kws363 PMC3649635

[17] Naik SS, Nidhi Y, Kumar K, Grover S. Diagnostic validity of premenstrual dysphoric disorder: revisited. Front Glob Womens Health. 2023;4:1181583. 38090047 10.3389/fgwh.2023.1181583 PMC10711063

[18] Bertone-Johnson ER, Whitcomb BW, Missmer SA, Manson JE, Hankinson SE, Rich-Edwards JW. Early life emotional, physical, and sexual abuse and the development of premenstrual syndrome: a longitudinal study. J Womens Health (Larchmt). 2014;23(9):729–39. 25098348 10.1089/jwh.2013.4674 PMC4158950

[19] Azoulay M, Reuveni I, Dan R, Goelman G, Segman R, Kalla C, et al. Childhood trauma and premenstrual symptoms: the role of emotion regulation. Child Abuse Neglect. 2020;108:104637. 32768748 10.1016/j.chiabu.2020.104637

[20] Morishita C, Inoue T, Honyashiki M, Ono M, Iwata Y, Tanabe H, et al. Roles of childhood maltreatment, personality traits, and life stress in the prediction of severe premenstrual symptoms. Biopsychosoc Med. 2022;16(1):11. 35484626 10.1186/s13030-022-00240-7 PMC9052504

[21] Gingnell M, Bannbers E, Wikström J, Fredrikson M, Sundström-Poromaa I. Premenstrual dysphoric disorder and prefrontal reactivity during anticipation of emotional stimuli. Eur Neuropsychopharmacol. 2013;23(11):1474–83. 24001875 10.1016/j.euroneuro.2013.08.002

[22] Baller EB, Wei SM, Kohn PD, Rubinow DR, Alarcón G, Schmidt PJ, et al. Abnormalities of dorsolateral prefrontal function in women with premenstrual dysphoric disorder: a multimodal neuroimaging study. Am J Psychiatry. 2013;170(3):305–14. 23361612 10.1176/appi.ajp.2012.12030385 PMC3968942

[23] Andreano JM, Cahill L. Menstrual cycle modulation of medial temporal activity evoked by negative emotion. Neuroimage. 2010;53(4):1286–93. 20637290 10.1016/j.neuroimage.2010.07.011 PMC3376005

[24] Rajkomar A, Dean J, Kohane I. Machine learning in medicine. N Engl J Med. 2019;380(14):1347–58. 30943338 10.1056/NEJMra1814259

[25] Lundberg SM, Erion G, Chen H, DeGrave A, Prutkin JM, Nair B, et al. From local explanations to global understanding with explainable AI for trees. Nat Mach Intell. 2020;2(1):56–67. 32607472 10.1038/s42256-019-0138-9 PMC7326367

[26] Steiner M, Macdougall M, Brown E. The premenstrual symptoms screening tool (PSST) for clinicians. Arch Womens Ment Health. 2003;6(3):203–9. 12920618 10.1007/s00737-003-0018-4

[27] Antonovsky A. Unraveling the mystery of health: How people manage stress and stay well [Kenko-no-nazo-wo-toku] Trans Yamazaki Y, Yoshii K. Tokyo: Yushindo Kobunsha; 1987.

[28] Parker G, Tupling H, Brown LB. A parental bonding instrument. Br J Med Psychol. 1979;52(1):1–10.

[29] Moos RH. The development of a menstrual distress questionnaire. Psychosom Med. 1968;30(6):853–67. 5749738 10.1097/00006842-196811000-00006

[30] Beck AT, Ward CH, Mendelson M, Mock J, Erbaugh J. An inventory for measuring depression. Arch Gen Psychiatry. 1961;4:561–71. 13688369 10.1001/archpsyc.1961.01710120031004

[31] Gaudry E, Vagg P, Spielberger CD. Validation of the state-trait distinction in anxiety research. Multivariate Behav Res. 1975;10(3):331–41. 26829634 10.1207/s15327906mbr1003_6

[32] The WHOQOL Group. Development of the World Health Organization WHOQOL-BREF quality of life assessment. Psychol Med. 1998;28(3):551–8. 9626712 10.1017/s0033291798006667

[33] Kato M. The development and standardization of Clinical Assessment for Attention (CAT) and Clinical Assessment for Spontaneity (CAS). Higher Brain Funct Res. 2006;26(3):310–9. (in Japanese).

[34] Chalder T, Berelowitz G, Pawlikowska T, Watts L, Wessely S, Wright D, et al. Development of a fatigue scale. J Psychosom Res. 1993;37(2):147–53. 8463991 10.1016/0022-3999(93)90081-p

[35] Hartmann JA, Carney CE, Lachowski A, Edinger JD. Exploring the construct of subjective sleep quality in patients with insomnia. J Clin Psychiatry. 2015;76(6):e768–73. 26132684 10.4088/JCP.14m09066

[36] Svedlund J, Sjödin I, Dotevall G. GSRS–A clinical rating scale for gastrointestinal symptoms in patients with irritable bowel syndrome and peptic ulcer disease. Dig Dis Sci. 1988;33(2):129–34. 3123181 10.1007/BF01535722

[37] Gross JJ, John OP. Individual differences in two emotion regulation processes: Implications for affect, relationships, and well-being. J Pers Soc Psychol. 2003;85(2):348–62. 12916575 10.1037/0022-3514.85.2.348

[38] Rosenberg M. Society and the adolescent self-image. Princeton: Princeton University Press; 1965.

[39] Agostini A, Rizzello F, Ravegnani G, Gionchetti P, Tambasco R, Ercolani M, et al. Parental bonding and inflammatory bowel disease. Psychosomatics. 2010;51(1):14–21. 20118436 10.1176/appi.psy.51.1.14

[40] Shibata M, Ninomiya T, Anno K, Kawata H, Iwaki R, Sawamoto R, et al. Perceived inadequate care and excessive overprotection during childhood are associated with greater risk of sleep disturbance in adulthood: The Hisayama Study. BMC Psychiatry. 2016;16:215. 27388724 10.1186/s12888-016-0926-2 PMC4936292

[41] Abbaspour A, Bahreini M, Akaberian S, Mirzaei K. Parental bonding styles in schizophrenia, depressive and bipolar patients: a comparative study. BMC Psychiatry. 2021;21(1):169. 33771132 10.1186/s12888-021-03177-3 PMC7995770

[42] Anno K, Shibata M, Ninomiya T, Iwaki R, Kawata H, Sawamoto R, et al. Paternal and maternal bonding styles in childhood are associated with the prevalence of chronic pain in a general adult population: The Hisayama Study. BMC Psychiatry. 2015;15:181. 26227149 10.1186/s12888-015-0574-y PMC4520085

[43] Hantsoo L, Epperson CN. Premenstrual dysphoric disorder: Epidemiology and treatment. Curr Psychiatry Rep. 2015;17(11):87. 26377947 10.1007/s11920-015-0628-3 PMC4890701

[44] Praschak-Rieder N, Willeit M, Winkler D, Neumeister A, Hilger E, Zill P, et al. Role of family history and 5-HTTLPR polymorphism in female seasonal affective disorder patients with and without premenstrual dysphoric disorder. Eur Neuropsychopharmacol. 2002;12(2):129–34. 11872329 10.1016/s0924-977x(01)00146-8

