# Supplementary material for: Association between maternal overprotection and premenstrual disorder: a machine learning based exploratory study
Source: Biopsychosoc Med. 2025 Feb 24;19:4. doi: 10.1186/s13030-025-00326-y (PMC11849209; doi:10.1186/s13030-025-00326-y)
Supplement: Supplementary file 1 — Additional file 1. Explanatory Variable Features. This file contains detailed information on the 443 explanatory variables. [file 13030_2025_326_MOESM1_ESM.docx]

**Additional File 1**

**Explanatory Variable Features**

A total of 443 explanatory variables are listed below.

**General questions**

The following 15 features were employed: age, phase in menstrual cycle, marital status, education level, age of menarche, menstrual cycle regularity, menstrual cycle, duration of menstruation, number of pregnancies, number of deliveries, use of female hormonal medications, sleep duration, menstrual pain severity, disturbance on daily life due to menstrual pain, and medication use for menstrual pain.

The following variables were treated as continuous: age, age at menarche, menstrual cycle, duration of menstruation, number of pregnancies, number of deliveries, and sleep duration. The following variables were treated as categorical variables: marital status, education level, menstrual pain severity, disturbance of daily life due to menstrual pain, and medication use for menstrual pain. Phase in menstrual cycle, menstrual cycle regularity, and use of female hormonal medications were also included as binary variables.

**Sense of Coherence**

A total of 14 features were employed: 13 items and a total score.

**Parental Bonding Instrument (PBI)**

A total of 56 features were employed: 25 items (PBIm: 1–25, PBIf: 1–25) for father and mother, respectively; a total score of each subitem of 'care' and 'overprotection', and quadrant classification.

**Menstrual Distress Questionnaire**

A total of 57 features were employed: 47 items, total score of nine subitems (pain, concentration, behavioral, autonomic, water retention, negative affect, arousal, control, and other), and total score.

**Beck Depression Inventory (BDI)**

A total of 22 features were employed: 21 items and total score.

**State-Trait Anxiety Inventory**

A total of 42 features were employed: 40 items and total score of two subitems (the State Anxiety Scale, and the Trait Anxiety Scale).

**Japanese version of the WHOQOL-BREF**

A total of 38 features were employed: 26 items, mean and total score of five subitems (physical health, psychological, social relationships, environmental health, Overall Quality of Life and General Health), and mean and total score.

**Clinical Assessment of Spontaneity questionnaire**

A total of 34 features were employed: 33 items and total score.

**Chalder Fatigue Scale**

A total of 15 features were employed: 14 items and total score.

**Pittsburgh Sleep Quality Index (PSQI)**

A total of 34 features were employed.

18 answers to each question: 1, 2, 3, 4, 5-a, 5-b, 5-c, 5-d, 5-e, 5-f, 5-g, 5-h, 5-j, 6, 7,8,9

16 types of scores in each component: Component 1 score, 2 sub-scores for Component 2, total score of 2 sub-scores of Component 2, Component 2 score, Component 3 score, Sleep efficiency (calculated by questions 1,3, and 4), Component 4 score, Sum of 5-b to 5-j scores, Component 5 score, Component 6 score, 2 sub-scores for Component 7, total score of Component 7, Component 7 score, and Global PSQI Score

**Gastrointestinal Symptom Rating Scale**

A total of 21 features were employed: 15 items, total score of five subitems (Regurgitation, Pain, Dyspepsia, Diarrhea, and Constipation), and total score.

**Emotional Regulation Questionnaire**

A total of 11 features were employed: 10 items and total score.

**Rosenberg Self-Esteem Scale**

A total of 11 features were employed: 10 items and total score.

**Parameters based on heart rate variability analysis**

A total of 73 features were employed:

The following data were extracted by “Kiritsu Meijin” (Crosswell Inc. Yokohama, Japan).

Activity: autonomic nervous system activity, used as five categorical variables

Reflection: Autonomic reflex, used as five categorical variables.

syn_type: The type of sympathetic nerves, used as seven categorical variables.

para_type: the type of parasympathetic nerves, used as five categorical variables.

meijin_score: Approximate overall rating calculated by “Kiritsu Meijin” (variable from 0 to 10 with 0.5 increments).

HR_rest_type: The type of heart rate at rest, used as five categorical variables.

HR_reflex_type: The type of heart rate at reflex, used as five categorical variables.

BP_rest_type: The type of blood pressure at rest, used as five categorical variables.

orthostatic_hypotension: Binary variables for presence of orthostatic hypotension.

sBP0: Binary variables for presence of orthostatic hypotension (systolic).

sBP1: Binary variables for presence of orthostatic hypotension (systolic, immediately after).

sBP2: Binary variables for presence of orthostatic hypotension (systolic, prolonged).

dBP0: Binary variables for presence of orthostatic hypotension (diastolic).

dBP1: Binary variables for presence of orthostatic hypotension (diastolic, immediately after).

dBP2: Binary variables for presence of orthostatic hypotension (diastolic, prolonged).

orthostatic_hypertension: Binary variables for presence of orthostatic hypertension.

data_stability_rest: Data stability in the resting sitting position (%).

data_stability_rise: Data stability when standing up (%).

data_stability_erect: Data stability in the standing position (%).

data_stability_sit: Data stability when sitting (%).

CVRR_rest: Coefficient of variation of R-R intervals at rest (%).

CVRR_rise: Coefficient of variation of R-R intervals at rise (%).

CVRR_erect: Coefficient of variation of R-R intervals in the standing position (%).

CVRR_sit: Coefficient of variation of R-R intervals in the sitting position (%).

d_CVRR_rise_rest: Difference between CVRR_rise and CVRR_rest (%).

ccvLperH_rest: √(Low Frequency) / √(High Frequency) / Mean R-R intervals at rest *100 (%)

ccvLperH_rise: √(Low Frequency) / √(High Frequency) / Mean R-R intervals at rise *100 (%)

ccvLperH_erect: √(Low Frequency) / √(High Frequency) / Mean R-R intervals in the standing position *100 (%)

ccvLperH_sit: √(Low Frequency) / √(High Frequency) / Mean R-R intervals in the sitting position *100 (%)

d_ccvLperH_rise_rest: Difference between ccvLperH_rise and ccvLperH_rest (%).

d_ccvLperH_erect_rise: Difference between ccvLperH_erect and ccvLperH_rise (%).

ccvHF_rest: √(High Frequency) / Mean R-R intervals in the resting *100 (%)

ccvHF_rise: √(High Frequency) / Mean R-R intervals at rise *100 (%)

ccvHF_erect: √(High Frequency) / Mean R-R intervals in the standing position *100 (%)

ccvHF_sit: √(High Frequency) / Mean R-R intervals in the sitting position *100 (%)

d_ccvHF_sit_erect: difference between ccvHF_sit and ccvHF_erect (%)

ccvHplusL_rest: √(High Frequency + Low Frequency) / Mean R-R intervals in the resting*100 (%)

ccvHplusL_rise: √(High Frequency + Low Frequency) / Mean R-R intervals at rise *100 (%)

ccvHplusL_erect: √(High Frequency + Low Frequency) / Mean R-R intervals in the standing position *100 (%)

ccvHplusL_sit: √(High Frequency + Low Frequency) / Mean R-R intervals in the sitting position *100 (%)

d_ccvHplusL_rise_rest: Difference between ccvHplusL_rise and ccvHplusL_rest.

ccvLF_rest: √(Low Frequency) / Mean R-R intervals in the resting *100 (%)

d_ccvLF_rise_rest: Difference between ccvLF_rise and ccvLF_rest.

d_ccvHF_erect_rise: Difference between ccvHF_erect and ccvHF_rise.

HR_rest: Heart rate in the resting sitting position.

HR_rise: Heart rate when standing up.

HR_erect: Heart rate in the standing position.

HR_sit: Heart rate when sitting.

sBP_rest: Systolic blood pressure in the resting sitting position.

sBP_rise: Systolic blood pressure when standing up.

BP_erect: Systolic blood pressure in the standing position.

sBP_sit: Systolic blood pressure when sitting.

mBP_rest: Mean blood pressure in the resting sitting position.

mBP_rise: Mean blood pressure when standing up.

mBP_erect: Mean blood pressure in the standing position.

mBP_sit: Mean blood pressure when sitting.

dBP_rest: Diastolic blood pressure in the resting sitting position.

dBP_rise: Diastolic blood pressure when standing up.

dBP_erect: Diastolic blood pressure in the standing position.

dBP_sit: Diastolic blood pressure when sitting.

HF_rest: High Frequency in the resting sitting position.

HF_rise: High Frequency when standing up.

HF_erect: High Frequency in the standing position.

HF_sit: High Frequency when sitting.

LF_rest: Low Frequency in the resting sitting position.

LF_rise: Low Frequency when standing up.

LF_erect: Low Frequency in the standing position.

LF_sit: Low Frequency when sitting.

LperH_rest: Low Frequency / High Frequency in the resting sitting position

LperH_rise: Low Frequency / High Frequency when standing up

LperH_erect: Low Frequency / High Frequency in the standing position

LperH_sit: Low Frequency / High Frequency when sitting

d_LperH_rise_rest: Difference between LperH_rise and LperH_rest.
