# Supplementary material for: Association between maternal overprotection and premenstrual disorder: a machine learning based exploratory study
Source: Biopsychosoc Med. 2025 Feb 24;19:4. doi: 10.1186/s13030-025-00326-y (PMC11849209; doi:10.1186/s13030-025-00326-y)
Supplement: Supplementary file 2 — Additional file 2. Searching for the optimal number of features. This file contains the figure used to explore the number of features via recursive feature elimination of the 443 features, to obtain the highest the area under the receiver operating characteristic curve (AUROC). [file 13030_2025_326_MOESM2_ESM.pptx]

## Slide 1
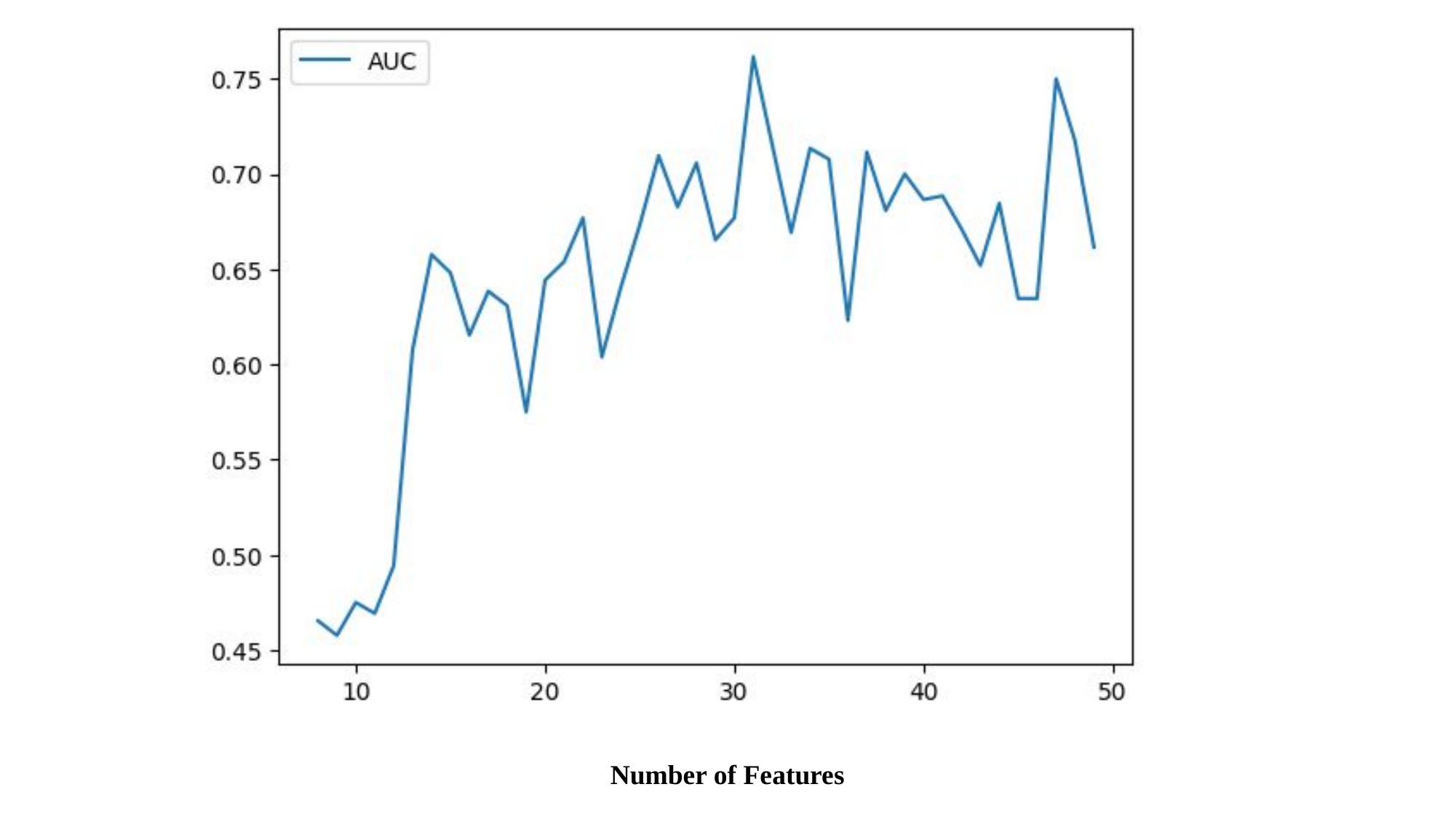

Number of Features

## Slide 2
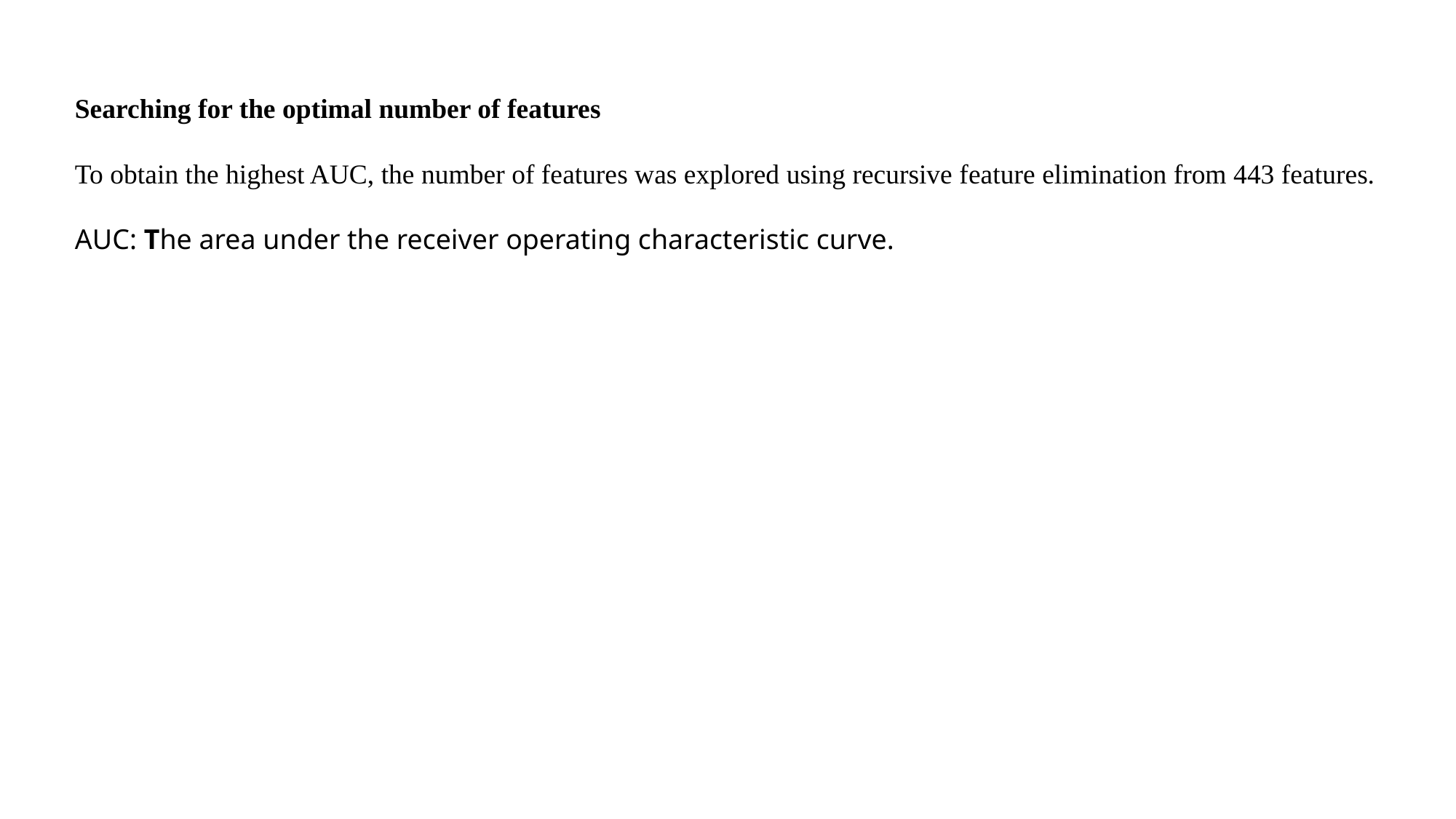

Searching for the optimal number of features
To obtain the highest AUC, the number of features was explored using recursive feature elimination from 443 features.
AUC: The area under the receiver operating characteristic curve.
