# Supplementary material for: Association between maternal overprotection and premenstrual disorder: a machine learning based exploratory study
Source: Biopsychosoc Med. 2025 Feb 24;19:4. doi: 10.1186/s13030-025-00326-y (PMC11849209; doi:10.1186/s13030-025-00326-y)
Supplement: Supplementary file 3 — Additional file 3. Selected 31 features. This file contains information describing the 31 explanatory variables selected by searching for the optimal number of features. [file 13030_2025_326_MOESM3_ESM.docx]

**Additional File 3**

**Selected 31 features**

'face7,' 'face8_3,' 'SOC9,' 'SOC11,' 'SOC_total,' 'PBIm_2,'

'PBIm_10,' 'PBIm_15,' 'PBIm_23,' 'PBIm_25,'

'PBIm_care,' 'PBIm_overprotection,' 'PBIf_1,' 'PBIf_23,'

'PBIf_care,' 'PBIf_overprotection,' 'd_CVRR_rise_rest,' 'ccvLF_rest,'

'sBP_rest,' 'sBP_erect,' 'sBP_sit,' 'mBP_rise,' 'dBP_erect,' 'dBP_sit,'

'LF_erect,' 'LF_sit,' 'MDQ_total,' 'STAI22,' 'STAI33,'

'QOL_social_total,' 'PSQI2_5c'
